# Climate change as an unexpected co-factor promoting coral eating seastar (*Acanthaster planci*) outbreaks

**DOI:** 10.1038/srep08402

**Published:** 2015-02-12

**Authors:** S. Uthicke, M. Logan, M. Liddy, D. Francis, N. Hardy, M. Lamare

**Affiliations:** 1 Australian Institute of Marine Science, PMB 3, Townsville MC, Townsville 4810, Queensland, Australia; 2 Schools of Medical and Biological Sciences, F13 - Anderson Stuart Building, University of Sydney, NSW 2006 Australia; 3 Department of Marine Science, University of Otago, Dunedin, New Zealand

## Abstract

Coral reefs face a crisis due to local and global anthropogenic stressors. A large proportion of the ~50% coral loss on the Great Barrier Reef has been attributed to outbreaks of the crown-of-thorns-seastar (COTS). A widely assumed cause of primary COTS outbreaks is increased larval survivorship due to higher food availability, linked with anthropogenic runoff . Our experiment using a range of algal food concentrations at three temperatures representing present day average and predicted future increases, demonstrated a strong influence of food concentration on development is modulated by temperature. A 2°C increase in temperature led to a 4.2–4.9 times (at Day 10) or 1.2–1.8 times (Day 17) increase in late development larvae. A model indicated that food was the main driver, but that temperature was an important modulator of development. For instance, at 5000 cells ml^−1^ food, a 2°C increase may shorten developmental time by 30% and may increase the probability of survival by 240%. The main contribution of temperature is to ‘push' well-fed larvae faster to settlement. We conclude that warmer sea temperature is an important co-factor promoting COTS outbreaks.

Coral reefs world-wide are under pressure because of global stressors such as warming sea surface temperatures (SST), ocean acidification^1^,^2^ and local disturbances such as eutrophication and overfishing^3^. These factors are often investigated in isolation, but the importance of interactions between local and global stressors is increasingly recognized and of greater relevance under future climate change scenarios^4^.

Coral cover on Australia's Great Barrier Reef (GBR) reduced by as much as 50% between 1985 and 2012, with a large proportion (~42%) of this decline attributed to outbreaks of the coral-eating seastar *Acanthaster planci* (crown-of-thorns-seastar, COTS)^5^ and coral bleaching (~10%) through increased SST. Outbreaks of COTS have been recorded on many Indo-Pacific coral reefs and are characterized by rapid adult population increases and associated massive loss of corals (reviewed in 6).

On the GBR, there are no indications of population outbreaks prior to the 1960's, although *Acanthaster* ossicles are present in sediments-layers older than 2000 yrs^7^. Three major primary outbreaks (1966, 1979, 1994) have been documented over the past ~50 yrs^8^,^9^,^10^. A fourth outbreak has recently (2011/12) commenced in the same area north of Cairns where previous outbreaks have started^11^.

Several factors are proposed as causes for COTS outbreaks, including reductions in predation pressure on juveniles and adults^12^, enhanced larval survival through reduced salinity^13^ and increased phytoplankton biomass (reviewed in^6^,^8^ ). The latter hypothesis, that increased phytoplankton through coastal runoff triggers COTS primary outbreaks is currently the most widely accepted, at least for the case of the GBR^6^. It is hypothesized that increased phytoplankton concentrations release larvae from food-limited development, which can shorten the development time of larvae^8^. This shortened development time will increase larval survival to settlement, as faster development will offset the high mortality rates thought to occur in the plankton (i.e. instantaneous mortality rates of echinoderm larvae are reported in the range of ≈0.10–0.20 day^−1^)^14^,^15^. Shorter larval duration may also reduce dispersion of pelagic larvae^16^, potentially localizing recruitment to a smaller area. This hypothesis hinges on the assumption that larvae in nature are food limited. Although previous experiments have compared larvae development under low food and higher food concentration^8^,^17^, no experiments have quantified developmental speed under a range of food concentrations with high replication, nor with temperature variation as a co-factor.

Positive effects of temperature on echinoderm development including asteroids, has been observed for species from polar to tropical habitats^18^,^19^,^20^,^21^. Development speed within thermal windows typically increases with warming due to the stimulating effects on physiological rates^22^,^23^,^24^. By contrast, physiological stresses at temperatures outside the species thermal window may decrease survival rates of marine developmental stages. Studies from intertidal areas showed that warm adapted species are more threatened by climate change as they already live close to their upper thermal limit^25^. This was also described for corals^26^ and is likely to apply to other coral reef invertebrates.

Temperature can affect the speed of embryonic and larval development in *A. planci*^21^, with larval development optimal at temperatures of about 28°C^13^,^27^. Johnson and Babcock^28^ also noted a narrowing of the temperature tolerance window of ≈1°C during development. The role of warmer temperatures in COTS outbreaks was alluded to by Johnson and Babcock^28^, who noted that *A. planci* outbreaks on the GBR initiate in the northern part of the distribution under higher SST, whereas fewer and smaller outbreaks occur in the more southern, cooler parts of the range.

Given that ocean warming and increased primary production may both benefit COTS larval development, it is important to understand the response of larvae when both factors are simultaneously applied; a situation that is likely to occur under realistic near-future ocean warming scenarios. In the present study, we hypothesize that increased food supply and a subtle increase in temperature may interact and accelerate larval development, and thus have the potential to reduce the time of the larval development cycle. In turn, this may decrease overall larval mortality in the plankton and lead to higher recruitment.

We found no evidence to reject this hypothesis. Strong additive to synergistic effects of increased microalgae concentration in the plankton and elevated SST on development suggest that runoff and climate change interactions may contribute to an increased frequency and/or amplitude of outbreaks.

## Results

### Development schedules

**Figure 1:**
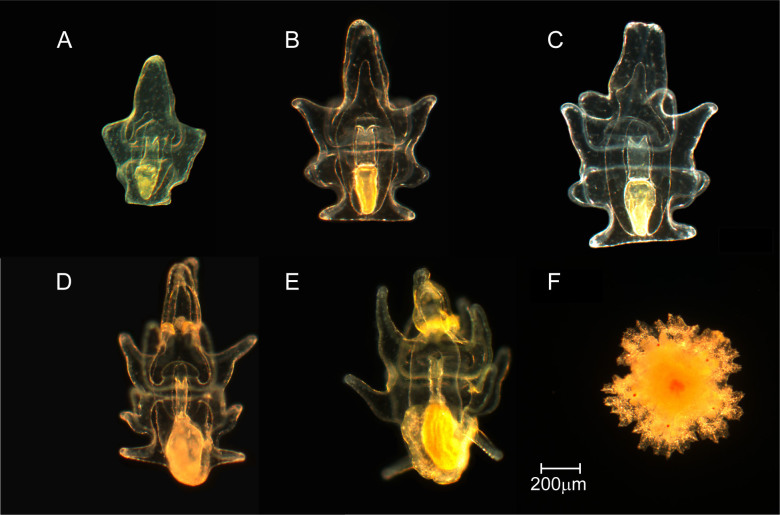
Developmental stages of *Acanthaster planci*. Stages shown are early and mid bipinnaria (A, B), early, mid and late brachiolaria (C–E), and a recently settled juvenile (F).

Culturing of *Acanthaster planci* gave rise to high quality larvae that followed the development schedule (Fig. 1) described by Keesing et al.^29^. Seastar planktotrophic development involves progeny progressing through an embryonic, bipinnaria and finally a brachiolaria stage prior to settlement^30^, with the majority of *A.planci* development time spent in the brachiolaria stage^17^. Here, we focus on development to mid-and late-stage brachiolaria larvae (MLB) combined as indicators for advanced development. In most cases, bipinnaria larvae (early development) and abnormally developing larvae exhibited the opposite trend to MLB (Supplementary Figure 1), with more favourable culture conditions (i.e. warmer temperatures, higher food concentration) enhancing the number of more advanced normal larva.

### Larval development and settlement

**Figure 2:**
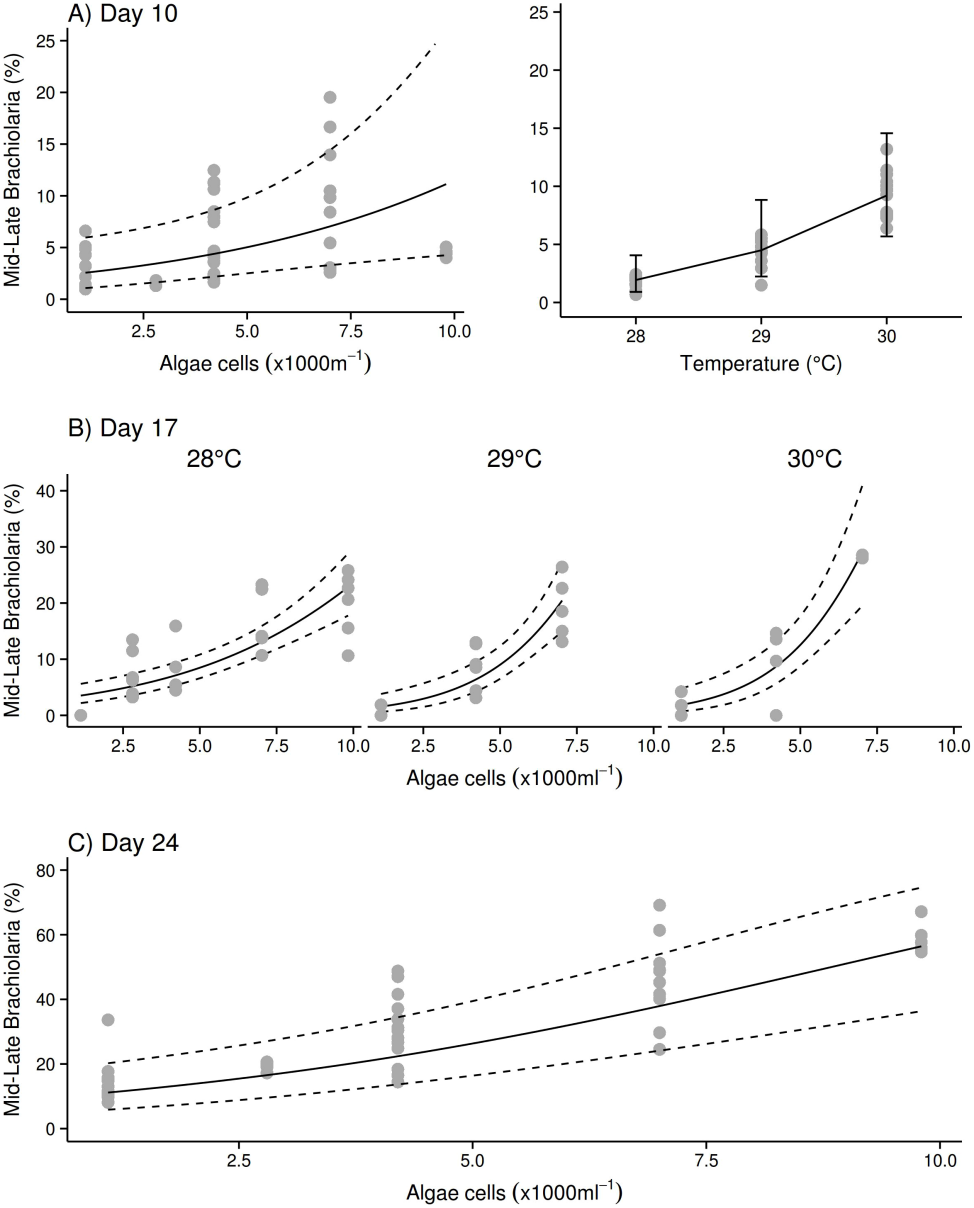
The effect of increased food and temperature on the percentage of late development *Acanthaster planci* larvae. Effects after 10d of development (A) are additive, and synergistic after 17d (B, significant interaction term present, see Table 1). After longer periods (C, 24d) low algae concentrations are still limiting development but temperature has no further enhancing effect. Dotted lines: 95% confidence interval or the fit.

At day 10 (7d after the start of feeding), there was an effect of algae and temperature in an additive fashion (GLM, Table 1), with more larvae at the MLB stages both under increasing temperature and food (algae) concentration (Fig 2A). For instance, a 2°C temperature increase lead to a 4.2–4.9 fold increase in MLB. Independent of food concentration, no late-stage brachiolaria were observed at 28°C on day 10. In contrast, the number of late-stage-brachiolaria, albeit small (<5%), clearly increased with food concentration at 29°C, and 30°C (Supplementary Fig. 1). Thus, some larvae under high food and elevated temperature treatments were near settlement competency after only 10d.

Fourteen days after the commencement of feeding (Day 17), there was an observable interaction between food supply and temperature (Table 1), indicating that the effects of both factors were synergistic at this point in time. An increase in both factors resulted in elevated numbers of MLB, but the level of response to higher food concentrations increased with higher temperature (slopes given in Supplementary Table 1). In fact, close inspection of the results (Fig. 2B) suggest that additional temperature effects on the percentage of MLB were mainly observed at higher food concentrations. For example, a 2°C temperature rise increased the percentage of MLB by a factor of 1.2 at 4000 algae cells ml^−1^; while at 9800 cells ml^−1^ the increase factor equated to 1.8 fold.

After 24d, the majority of larvae in the higher food concentrations reached mid- or late-brachiolaria stage (Supplementary Fig. 1). At the lowest food concentrations, ~10% mid- brachiolaria were also present, but less than 5% were at late-brachiolaria stage. The effect of food concentration on MLB remained highly significant at Day 24, but temperature had no further accelerating effect (Table 1). From Day 20, onward, larvae started to settle in low numbers inside the experimental containers of most treatments without providing settlement inducers, with the exception of all replicates under the two lowest algae concentrations. The experiment was stopped on Day 24, but we kept the lowest two food concentrations until Day 35, at which stage settlement was still not observed (Supplementary Table 2). On Day 24, settlement could be induced in larvae from all treatments except the lowest 2 food concentrations (Supplementary Table 2).

**Figure 3:**
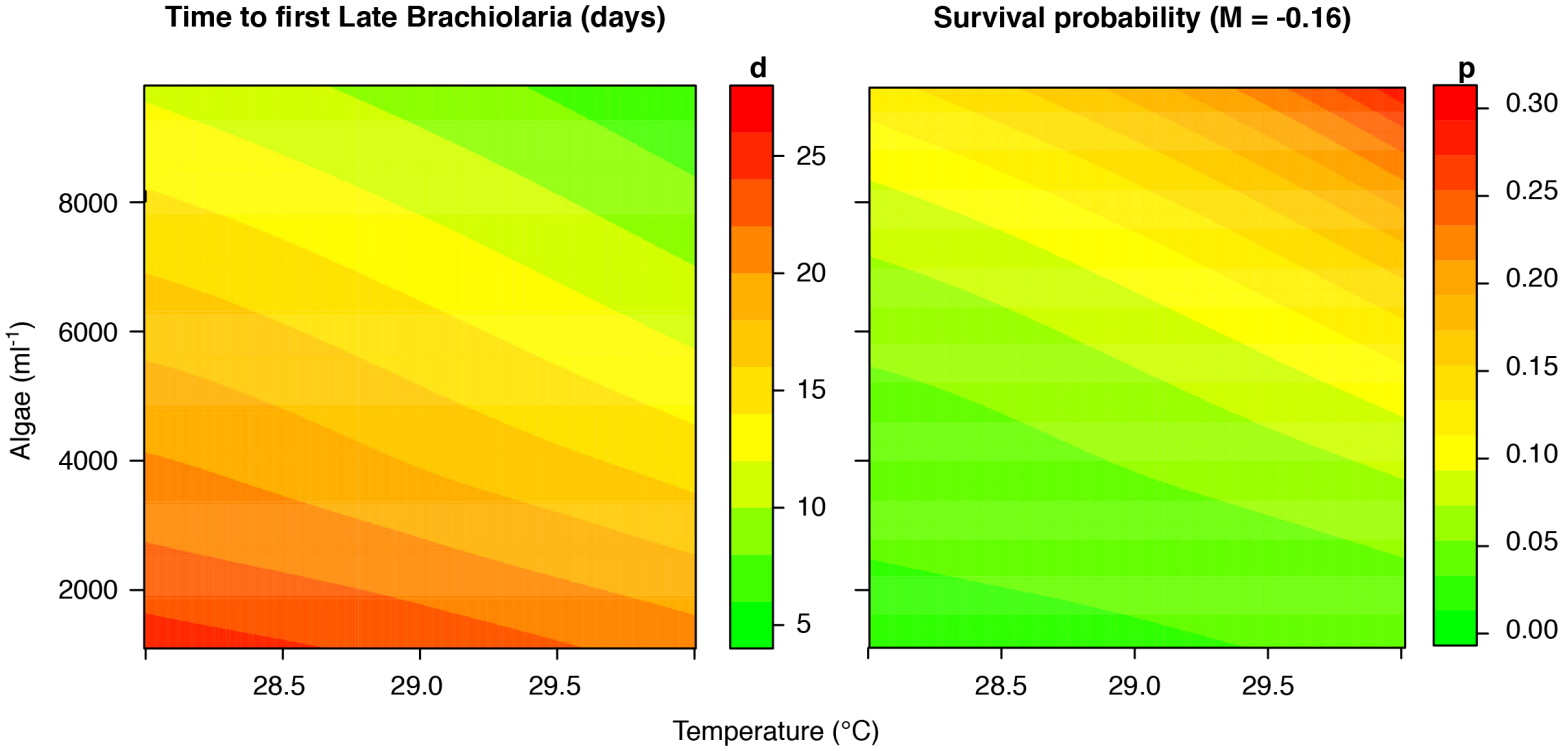
Level plots (based on LOESS fitting) illustrating the combined effects of food (algal cells ml^−1^) and increased temperature on the speed of development of *Acanthaster planci* larvae (A). B) Illustrates the effect of faster development and resulting shorter planktonic developmental time. The survival probability for the fastest developing larvae under each treatment were calculated assuming a daily mortality rate of M = −0.16.

To further illustrate the combined effect of food availability and temperature on the speed of larval development, we fitted a LOESS smoother through data on the time the first competent larvae in each treatment were observed. A level plot of that model highlighted how the time to reach competency was shortened with higher food concentrations (Fig. 3A), and that temperature increase in the range investigated further accelerated developmental speed. The resulting survival probability to settlement for the fastest larvae (Fig. 3B) exhibited the same pattern. For instance, at 5000 cells ml^−1^ food, a 2 °C increase in temperature may shorten developmental time from 18.8 d to 13.2 d (~30%). Under the assumption of an instantaneous mortality rate of 0.16 day^−1^, this equates to an increase in survival probability of the fastest larvae from 3.6 to 8.7%, a 2.4-fold increase.

### Larval morphology

**Figure 4:**
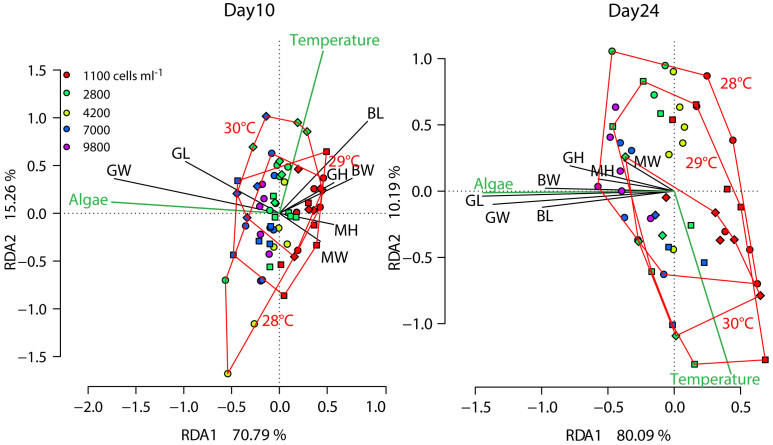
Biometric analyses of *Acanthaster planci* larvae at day 10 and 24 of their development under different algal concentrations and temperatures. Food concentrations are indicated by colour (see legend) and samples from the same temperature are surrounded by red outlines. In addition, temperature treatments are indicated by different symbols (28°C: circles, 29°C: squares, 28°C: diamonds). Black vectors are the individual biometric measures (BL: Body Length, BW: Body Width, GH: Gut Hood, GL: Gut Length, GW: Gut Width, MH: Mouth Hood, MW: Mouth Width) and green vectors represent the environmental variables. Analyses and plots are based on average values per replicated experimental container.

Biometric measures of larvae were taken on Days 10 and 24. Seven days after feeding, both temperature (permutation analysis: p = 0.032) and food concentration (p < 0.001) explained a significant amount of the variation of larval shape in a redundancy analysis (Fig. 4). The amount of variance explained by temperature was small (5.25%), and the temperature vector was correlated to larvae with longer bodies. By contrast, food explained a higher amount of variance (16.26%), and at Day 10 higher food concentrations were associated mainly with longer and wider guts (Supplementary Fig. 2).

After 24d, the effect of temperature was non-significant (p = 0.100) in the redundancy analysis, and no morphometric measures correlated with that factor (Fig. 4). By contrast, food concentrations remained a significant environmental variable (P < 0.001) and explained a large amount of the variance in larval morphology observed (47.48%). After three weeks of feeding, all biometric measures were positively correlated to food supply. Thus, higher food concentration resulted in larger larvae (Supplementary Figure 3). Similar to the analysis of the development stages (see above), we propose that the effects of temperature were reduced at this late stage of development because larvae at lower temperatures have now ‘caught up' with development. In addition, settlement of the largest larvae in higher temperatures and potentially mortality will have selectively removed larger larvae.

### Larval mortality

On day 24, larval densities remained high (global average: 0.45 larvae ml^−1^, SD = 0.24 larvae ml^−1^), with daily mortality rates (average M ≈ −0.07 day^−1^) resulting in a density of 0.04–0.31 larvae ml^−1^ after 24d of culturing. A higher mortality was observed in the highest temperature treatment, with 8 out of 18 replicates in that temperature experiencing high mortality. Given that larvae in the remaining replicates of the same temperature did well, we attributed the loss to husbandry issues [i.e. bacterial infections more likely in higher temperatures, e.g.^31^] and not to a direct adverse effect of elevated temperature on larval physiology.

## Discussion

Our experiments illustrated that there are important additive to synergistic effects of food concentration and elevated sea surface temperature on developmental pace of *Acanthaster planci* larvae, while the size of the larvae was mainly determined by food concentrations. Modeling these interactions illustrated how change in both local (eutrophication) and global factors (ocean warming) may interact in the future to shorten larval development, with a likely consequence being an increase in settlement and enhancing the probability of COTS outbreaks. The Great Barrier Reef (GBR) is currently suffering its 4^th^ outbreak of *Acanthaster planci* since the 1960, and predation of this seastar has substantially contributed to the 50% loss in coral cover since 1985^5^. Enhanced larval survivorship through episodically increased phytoplankton biomass is presently the most parsimonious hypothesis to explain primary outbreaks in the area north of Cairns where all outbreaks initiate^8^,^32^. However, due to a lack of quantitative studies on several aspects of larval ecology, such as the role of temperature, there is still some debate about the validity of this hypothesis^11^. It is most likely that the subsequent spread of secondary outbreaks (usually southward) results from massive larval production through adults in the primary outbreaks, where denser adult populations overcome any allele effects and achieve very high fertilization rates through higher sperm concentration and resulting higher sperm : egg ratios^33^,^34^.

A positive role of elevated food concentrations and warmer temperatures is established for larval development and survival in a range of invertebrates, including faster development in response to higher food levels in echinoids^35^,^36^,^37^,^38^ and asteroids^39^,^40^,^41^,^42^, and to warmer temperatures in both classes^19^,^21^. The increases in the developmental rate of *Acanthaster* larvae in response to both variables observed in the present study are consistent with previous experiments on the species in which the two factors were considered separately. Temperature influences larval development in *A. planci*^21^ with larval development fastest at temperatures ≈28°C^13^,^27^. Early-stage *Acanthaster* larvae from the GBR have a well-defined thermal window between 24 and 32°C, that reflects their spatial distribution limited to locations that have summer temperatures within this range^28^,^43^. The latter study also noted that within this range (and specifically for the present findings, between 28 and 30°C) there was no significant change in the size of larvae or abnormality rates, while there was an increase in development rate through the early embryological and larval stages.

The positive response of *Acanthaster* larvae to elevated food found here was first quantified by Lucas^17^, with the significance of these observations recognized by a number of researchers^9^,^17^,^32^ as one hypothesis for the timing of COTS outbreaks due to relaxation of larval starvation. The hypothesis was tested and modeled by Fabricius et al.^8^, who noted the proportion of larvae completing development was negligible at low food concentration (at that study <0.5 μg Chl-a L^−1^), but increased when food was elevated to 2.0 μg Chl-a L^−1^. Chlorophyll-a concentration also influenced body length in the latter study. When we consider the results of the present study for food concentration only (i.e. results standardized to intermediate temperatures), we observed a 5-fold increase in the presence of late-brachiolaria (i.e. those completing development) at 24 days across our range of food concentrations (<1000 cells ml^−1^ to ≈10,000 cells ml^−1^). Across the same algal concentration range we also noted an average increase in body length and width of ≈30% and 20%, respectively. A direct comparison of our development rates using chlorophyll-a concentration given in Fabricius et al.^8^ are difficult, given that we used a different algae composition with species-specific chlorophyll contents, while even the same algal strains can contain different cell specific chlorophyll concentrations. Even summer chlorophyll concentrations on inshore reefs of the GBR are generally below 0.5 μg Chl-a L^−1 32^, but concentrations over 2 μg Chl-a L^−1^ which increased larval development can be triggered by nutrient runoff through flood-plumes following extreme rain events^44^. Our experimental algal cell densities for *Dunaliella* and *Phaeodactylum* were within the range previously used for *A. planci* larval feeding experiments^17^,^45^ , but no data exists for *Chaetocerus*. Although there is limited information on algal cell numbers on the GBR, numbers >1000 cells ml^−1^ are rare, but occur after flood events^46^.

Previous studies provided some evidence for a key role of larval development and survival in driving COTS outbreaks, but do not assess the role of temperature in the process. In fact, few larval studies have simultaneously examined the interaction of food concentration and water temperature on larval development. Laboratory experiments demonstrated an additive effect of food and temperature increase in barnacles such as *Balanus albiocastatus*^47^ and *Balanus amphitrite*^48^. Meekan et al.^49^ used environmental data to explore the importance of temperature and food on growth in natural occurring late-stage fish larvae, and concluded temperature explained more of the observed variation in growth.

Our observations suggest that an important interactive effect of temperature on the response of larvae to food concentrations can occur, with warming enhancing the response to food concentrations. The most important enhancement appeared in the first 1–2 weeks after commencement of feeding. In that period, increased temperatures significantly boosted the positive effect of high food concentrations by further reducing developmental times.

Such observations provide an insight into the response of *Acanthaster* larvae to spatial and temporal variations in both sea temperature and food concentrations observed on the GBR. For example, *Acanthaster* has a distribution that ranges from 24 to 32°C^43^, and therefore a response to higher natural food levels could be greater in the warmer part of the species range. Indeed, COTS primary outbreaks typically occur in the warmer, northern parts of the species range^8^ with secondary outbreaks generally spreading south. Hoegh-Guldberg and Pearse^21^ showed the time required to reach hatching increased between 20 and 32°C, with a breakpoint at 25°C. Lamare et al.^43^ found the thermal window for development until early brachiolaria stage to be 25.6 to 31.6°C, with an optimum at 28.7°C, slightly above current temperatures during the spawning season in the study area. Thus, a slight SST increase may increase COTS development, although, one other study has observed 100% mortality of larvae at 30°C^50^.

The role of temporal variation in SST is less clear. Although electronic temperature logger records only cover the last two outbreaks (http://data.aims.gov.au/aimsrtds/datatool.xhtml) we could find no direct correlation between COTS outbreaks and above-average temperature years (data not shown). However, average sea temperatures of the GBR have already increased by approximately 0.6°C^51^ (updated data, J. Lough pers. comm) over the last 130 yrs and are expected to further increase 1–2 degrees by 2070 under low to moderate emission scenarios^52^. Thus, it is likely that the boosting effect described here already occurs when increased average temperatures during the spawning season coincide with elevated algae concentrations in years with high runoff, an effect that may further increase in the future.

In addition to development rates, levels of mortality prior to settlement will also drive levels of recruitment. Although survival probabilities modelled here were based on mortality rates derived for temperate sea urchins because they were the best available data^14^,^15^, this is sufficient to illustrate the outcome of a shorter larval life on numbers reaching settlement. For instance survival probability of the fastest larvae increased by ~270% when increasing food from 1100 to 5000 cells ml^−1^ at 28°C. Increasing temperature by 2°C at the higher algae concentration elevates survival by a further 240%. These numbers are somewhat below the 8-fold increase in modelled by Fabricius et al.^8^ when chlorophyll-a concentrations were doubled. This difference is mainly due to our model only predicating survival probabilities of the fastest larvae. In reality, hardly any larvae reached late stage brachiolaria in the low food concentrations, and no larvae could be settled in the two lowest food concentrations until Day 35, when the experiment ended.

Greater numbers of larvae reaching settlement under warmer and more productive GBR waters would likely alter the number of recruits entering *Acanthaster* populations. *A. planci* larvae show a high degree of settlement preference^34^,^53^, with competent larvae thought to selectively settle on specific crustose coralline algae associated with coral reefs. Post-settlement survival of *Acanthaster* appears age-specific, decreasing from 6.49% day^−1^ in 1 month old juveniles to 0.45% in 7-month old juveniles, with survival thought to be predator-limited^54^. While it is difficult to directly link our experimental findings to population level changes, if *A. planci* populations are recruitment limited^6^ as is assumed for most coral reef animals^55^, then the preferential settlement on coral reefs of large cohorts of larvae that can quickly growth through initially low post-settlement survival, supports a mechanism whereby enhanced pre-settlement development would directly increase the number of adults.

In conclusion, our study confirmed that COTS larvae do not reach settlement stage below a certain food threshold and that over a range of algae concentrations developmental speed increases with food supply. We also showed that an environmentally relevant temperature increase can further enhance developmental speed and lead to a higher percentage of larvae rapidly reaching settlement stage, thus increasing the number of total settlers. To conclude that developmental acceleration is sufficient for temperature to act as a significant co-contributor in outbreaks mainly depends on mortality rates assumed for population models. We applied 16% mortality rate per day, although daily marine larval mortality rates can vary between 2 to 100%^14^. Thus, while much focus has been on quantifying development rate in response to environmental change, it is equally important that realistic larval mortality rates are established for *Acanthaster planci*. Further testing the function of climate change and land runoff in the role of promoting COTS outbreaks will also require detailed considerations of nutritious quality of different micro-algal species. This needs to be accompanied by studies establishing which algae species increase under elevated land-runoff.

## Methods

### Specimen collection and spawning

Adult *Acanthaster planci* specimens were collected early November 2013 on Agincourt reef (Cairns section of the Great Barrier Reef, Australia, 16°01.2′S, 145°51.1′E), transported to the Australian Institute of Marine Science and kept in natural seawater under flow through conditions.

For fertilization, a small (≈1 cm) incision was made near the proximal end of one of the arms and 3–4 gonadal lobes were removed from each individual. Gonads were sexed and testes of six males and ovaries of six females where collected. Testes were placed in covered 6-well plates to prevent desiccation. Ovary lobes were rinsed with filtered seawater to remove loose eggs and were subsequently submerged in a 10^−5^ M 1-methyladenine/seawater solution to induce maturation and egg release from the lobes. After 60–70 min, mature eggs that sunk to the bottom of the beaker were washed through a 500 μM mesh, and eggs from all females combined resulting in a stock solution of 400 eggs mL^−1^. Two μl of sperm from each male was combined and added to 2500 ml of the egg solution, resulting in a concentration at fertilization of 10^6^–10^7^ sperm ml^−1^ which yielded >99% fertilization. After 20 min, eggs were washed repeatedly using a 50 μm mesh to remove excess sperm. Subsequently, eggs were diluted evenly in six 70 L tanks, 2 at each of the three experimental temperatures. Larvae were washed after 24 h and kept under the same conditions for a total of 72 h, the time when larvae usually start feeding^29^.

Each of 6 treatment jars for each algae and temperature combination were stocked with 2500 larvae ( = 1 larva ml^−1^) from the respective temperature treatments. Cleaning of the jars and 100% water replacement was made every second day by carefully washing larvae over a 200 μm mesh. Densities and scoring of the larval stages (see Fig. 1) was conducted on days 10, 17 and 24 of the experiment, with samples for biometric measurements collected on days 10 and 24. Prior to photography, larvae were concentrated, relaxed in 6.8% MgCl_2_ then fixed in 4% paraformaldehyde. Larvae (>20 per replicate) were photographed on a Zeiss Axioscope at 50 times magnification. Measurements of the larvae followed procedures described in^43^ with the length of the aboral hood, oral hood, body length, body width, stomach width, stomach length recorded. Measurements were analyzed in ImageJ^56^ after calibration.

### Feeding

We cultured three algae types from pure strains supplied by Australian Algae Culture Collection (Hobart). *Chaetoceros* sp. (Strain No CS-256), *Phaeodactylum tricornutum* (CS-29), and *Dunaliella* sp. (CS-353). Although both *Phaeodactylum* and *Dunaliella* were previously used as single feeds to raise *Acanthaster* larvae^17^,^57^, we decided to feed a mix of algae to provide different nutritional sources. In addition, the former two strains are algae occurring in the GBR providing higher ecological relevance to the food sources. Algae were fed in equal proportion with regards to cell-specific chlorophyll content measured in the cultures (*Chaetocerus*: 3.83 10^−7^ μg cell^−1^, *Phaeodactylum*: 1.91 10^−7^ μg cell^−1^, *Dunaliella*: 1.56 10^−6^ μg cell^−1^). Thus, with regards to cell numbers only 8% of the algae fed were *Dunaliella*, 61% *Phaeodactylum* and 31% *Chaetocerus*. Based on previous studies^8^,^17^, in the 28°C treatment we applied 5 food concentrations with the aim to cover a range from severely food limited to fully satiated (1100, 2800, 4200, 7000 and 9800 cells ml^−1^). The remaining two temperature treatments were stocked with 1100, 4200 and 7000 cells ml^−1^.

Algae were cultured in F/2 medium with silicate added. Cultures were kept at 24°C at a 12:12 light dark cycle. Medium was prepared in 0.2 μm filtered and autoclaved (120°C, 20 min) seawater. In order to feed the larvae at the target concentrations algal cells of the three cultures were counted five times week^−1^ on a haemocytometer and respective algal concentrations calculated. Chlorophyll samples (100 ml) were taken on four occasions from three representative culture vessels of each algae concentration and filtered over 0.45 μm GFC filters. Filters were ground in 90% acetone, and subsequently, chlorophyll-a was measured fluorometrically^58^. Chlorophyll results are given in Supplementary Table 3.

### Experimental design

Experimental units were 2 L glass jars, with plastic lids allowing aeration through a Perspex pipe. We manipulated water to three temperatures tightly controlled by heat exchangers and submerging the experimental chambers in flow-through water jackets (Supplementary Figure 4). Temperatures in one representative per treatment were recorded every minute (N = 39399) using Hoboware temperature loggers. We targeted temperatures 28°C (average: 27.83°C, SD = 0.13°C), 29°C (average: 28.75°C, SD = 0.05°C) and 30°C (average: 29.81°C, SD = 0.05°C), representing present day average sea temperatures in the source area of the adults during larval development on the GBR (~November to December), one and two degrees SST increase respectively details of water temperature during larval development time see:^43^.

### Statistical analyses

Data for development consisted of percentages of individual larval stages for each replicate treatment jar (N = 6). We used generalized linear models (GLM:^59^) with quasi-binomial link functions to test for the effect of algae concentration and seawater temperature on larval stages. These models are most appropriate to fit percentage data because the fit is made as log-odds ratios. This type of model is also robust to unbalanced designs^60^, and to further accommodate this we used marginal sums of squares. Algal concentrations were used as a continuous factor, while temperature was used as a categorical factor because it only had three factor levels. Initial models included an interaction between algae concentration and temperature, however, interaction terms were subsequently removed when p > 0.25^60^.

Redundancy Analysis (RDA:^61^) was used to test for the effect of the environmental variable (Algae, Temperature) on the morphology of the larvae. To achieve this, we z-transformed (mean = 0, SD = 1) the morphometric measures and used averages per experimental replicate to avoid pseudo-replication. Whether environmental factors explained a significant amount of the variation in the data was tested using permutation tests (10,000 permutations).

We estimated the probability of survival for larvae based on the first presence of competent late-stage brachiolaria larvae with well-developed rudiments in each individual treatment. Based on previous experience with settlement, we assumed that these competent larvae could settle within 2 days. Estimates for mortality rates in echinoderms with similar larval length and ecology (free spawned feeding larvae) are available only for temperate sea urchins^14^,^15^. Thus, we used the average value (M = −0.16) of four species published as indicative planktonic mortality rate. The occurrence of first brachiolaria and the resulting estimates for survival probability of the fastest larvae at the respective algae concentrations and temperatures were fitted using LOESS (local polynomial regression) smoothing data presented in contour plots.

## Author Contributions

S.U., D.F. and M.L. conducted experiment, analysed data and wrote the MS, M.L. assisted with statistical analysis and edited the MS, M.L. and N.H. analysed samples and data and maintained the experiment.

## Supplementary Material

Supplementary Information Supplementary Tables and Figures

## Figures and Tables

**Table 1:** Analysis of deviance for the generalized linear models testing the effects of food concentration and temperature on the percentage of *Acanthaster planci* larvae developed to mid- and late-brachiolaria stage at three measurement days. Interaction terms were highly insignificant on D10 (0.4920) and D 24 (p = 0.4084) and were thus removed from the models. Marginal sums of squares were used to accommodate the unbalanced sampling design

	χ^2^	DF	P
D10			
Algae	6.63	1	0.0100
Temperature	14.13	2	0.0002
D17			
Algae	105.54	1	<0.0001
Temperature	3.31	2	0.0452
Algae x Temperature	5.81	2	0.0056
D24			
Algae	29.59	1	<0.0001
Temperature	0.01	2	0.9311
