# Supplementary material for: Climate change as an unexpected co-factor promoting coral eating seastar (Acanthaster planci) outbreaks
Source: Sci Rep. 2015 Feb 12;5:8402. doi: 10.1038/srep08402 (PMC4325318; doi:10.1038/srep08402)
Supplement: Supplementary Information — Supplementary Tables and Figures [file srep08402-s1.doc]

**Supplementary Information: Climate change as an unexpected co-factor promoting coral eating seastar (*Acanthaster planci*) outbreaks**

S. Uthicke, M. Logan, M. Liddy, D. Francis, N. Hardy, M. Lamare

Supplementary Figure 1: The percentage of abnormal larvae (Abnormal), Bipinnaria, Early Brachiolaria, Mid Brachiolaria, Late Brachiolaria and larval densities of *Acanthaster planci* at 3 days during the experiments, different colours indicate temperature treatments (Blue = 28°C, Green = 29°C and Red = 30°C). Please note that some symbols are hidden behind others.

Supplementary Figure 2: Biometric measures showing some degree of correlation with Temperature (top two panels) or food concentration (bottom two panels) in the redundancy analysis of D10. Box and whisker plots are based on average values per replicated (experimental container). Whiskers denote 1.5 x the inter-quartile range, the black line indicates the mean.

Supplementary Figure 3: The relationship of food concentrations on seven biometric measures of *Acanthaster planci* larvae at D24. All measures also showed some degree of correlation to food concentrations in the redundancy analysis. Box and whisker plots are based on average values per replicated (experimental container). Whiskers denote 1.5 x the inter-quartile range, the black line indicates the mean.

Supplementary Figure 4: Temperature profiles for the three temperature treatments referred to as 28°C (average: 27.83°C, SD = 0.13°C), 29°C (average: 28.75°C, SD = 0.05°C) and 30°C (average: 29.81°C, SD = 0.05°C). Temperatures were recorded every minute (N = 39399) using Hoboware temperature loggers.

Supplementary Table 1. Details of the slopes and intercepts of generalized linear models testing the effects of food concentrations and temperature on the percentage of *Acanthaster planci* larvae developed to mid and late brachiolaria stage at three measurement days.

|  | Estimate | SE | t | p |
| --- | --- | --- | --- | --- |
| D 10 |  |  |  |  |
| Intercept | -4.704 | 0.587 | -8.01 | <0.0001 |
| Algae | 0.179 | 0.072 | 2.50 | 0.0152 |
| Temperature 29 | 0.869 | 0.519 | 1.68 | 0.0993 |
| Temperature 30 | 1.637 | 0.457 | 3.58 | 0.0001 |
| D 17 |  |  |  |  |
| Intercept | -3.580 | 0.280 | -12.80 | <0.0001 |
| Algae | 0.241 | 0.037 | 6.52 | < 0.0001 |
| Temperature 29 | -1.088 | 0.617 | -1.76 | 0.0845 |
| Temperature 30 | -0.957 | 0.653 | -1.47 | 0.1494 |
| Algae x Temperature 29 | 0.232 | 0.098 | 2.37 | 0.0219 |
| Algae x Temperature 30 | 0.280 | 0.110 | 2.54 | 0.0146 |
| D 10 |  |  |  |  |
| Intercept | -2.054 | 0.359 | -5.72 | <0.0001 |
| Algae | 0.268 | 0.053 | 5.07 | < 0.0001 |
| Temperature 29 | -0.315 | 0.359 | -0.88 | 0.3850 |
| Temperature 30 | 0.075 | 0.400 | 0.19 | 0.8520 |

Supplementary Table 2 Summary of additional observations during the feeding and temperature experiment on *Acanthaster planci* at each of the temperature and algae treament combinations. Un-induced settlement refers to settlement and metamorphosis in the experimetnal containers. Induced settlement refers to a trial on D24, offering *Lithothamnium pseudosorum* in 6 well containers . + and – refers to the presence or absence of metamorphosed larvae after 24h. *Lithothamnium pseudosorum* is the preferred settlement substrate for *A. planci* larvae

| Temperature  (°C) | Algae  (cells ml^-1^) | First Late Brachiolaria  (days) | First un-induced settlement  (days) | Induced settlement  (D 24) |
| --- | --- | --- | --- | --- |
| 28 | 1100 | 24 | >35 | - |
|  | 2800 | 24 | >35 | - |
|  | 4200 | 17 | 24 | + |
|  | 7000 | 17 | 20 | + |
|  | 9800 | 10 | 22 | + |
| 29 | 1100 | 24 | >35 | - |
|  | 4200 | 17 | 24 | + |
|  | 7000 | 17 | 20 | + |
| 30 | 1100 | 24 | >35 | - |
|  | 4200 | 10 | 20 | + |
|  | 7000 | 10 | 20 | + |

Supplementary Table 3: Algae concentrations (in cell numbers ml^-1^), consisting of 8% *Dunialiella*, 62% *Phaeodactylum* and 31% *Chaetocerus,* and measured chlorophyll concentrations in the treatment containers. Given is the average of 4 measuring days, the standard deviation is given in brackets. The average chlorophyll concentration is significantly related to the target number of algae (R^2^ = 0.99, p < 0.001).

| Algae | Chlorophyll (µg l^-1^) |
| --- | --- |
| 1100 | 0.67 (0.31) |
| 2800 | 1.80 (0.90) |
| 4200 | 2.55 (1.07) |
| 7000 | 4.85 (1.33) |
| 9800 | 7.11 (1.18) |

Supplementary references

Johnson, C. R., D. C. Sutton, R. R. Olson, and R. Giddings. 1991. Settlement of crown-of-thorns starfish: role of bacteria on surfaces of coralline algae and a hypothesis for deepwater recruitment. *Mar. Ecol. Prog. Ser.* **71**:143-162.

Uthicke, S., D. Pecorino, R. Albright, A. P. Negri, N. Cantin, M. Liddy, S. Dworjanyn, P. Kamya, M. Byrne, and M. Lamare. 2013. Impacts of Ocean Acidification on Early Life-History Stages and Settlement of the Coral-Eating Sea Star *Acanthaster planci*. *PLoS ONE* **8**:e82938.
